# Extracellular Vesicle-Mediated Immune Regulation of Tissue Remodeling and Angiogenesis After Myocardial Infarction

**DOI:** 10.3389/fimmu.2018.02799

**Published:** 2018-11-29

**Authors:** Santiago Sánchez-Alonso, Ana Alcaraz-Serna, Francisco Sánchez-Madrid, Arantzazu Alfranca

**Affiliations:** ^1^Immunology Service, Hospital de la Princesa, Instituto de Investigación Sanitaria del Hospital Universitario de La Princesa, Universidad Autónoma de Madrid, Madrid, Spain; ^2^Department of Vascular Biology and Inflammation, Centro Nacional de Investigaciones Cardiovasculares, Madrid, Spain; ^3^CIBER Cardiovascular, Madrid, Spain

**Keywords:** small EVs, tissue remodeling, myocardial infarction, immune system, angiogenesis

## Abstract

Myocardial ischemia-related disorders constitute a major health problem, being a leading cause of death in the world. Upon ischemia, tissue remodeling processes come into play, comprising a series of inter-dependent stages, including inflammation, cell proliferation and repair. Neovessel formation during late phases of remodeling provides oxygen supply, together with cellular and soluble components necessary for an efficient myocardial reconstruction. Immune system plays a central role in processes aimed at repairing ischemic myocardium, mainly in inflammatory and angiogenesis phases. In addition to cellular components and soluble mediators as chemokines and cytokines, the immune system acts in a paracrine fashion through small extracellular vesicles (EVs) release. These vesicular structures participate in multiple biological processes, and transmit information through bioactive cargoes from one cell to another. Cell therapy has been employed in an attempt to improve the outcome of these patients, through the promotion of tissue regeneration and angiogenesis. However, clinical trials have shown variable results, which put into question the actual applicability of cell-based therapies. Paracrine factors secreted by engrafted cells partially mediate tissue repair, and this knowledge has led to the hypothesis that small EVs may become a useful tool for cell-free myocardial infarction therapy. Current small EVs engineering strategies allow delivery of specific content to selected cell types, thus revealing the singular properties of these vesicles for myocardial ischemia treatment.

## Introduction

Regenerative medicine is focused on repairing damaged or malfunctioning tissue through both cellular and non-cellular therapies, such as tissue engineering or treatments based on biomaterials. This biological strategy is applicable to a wide spectrum of processes, including wound healing (1), neurological conditions (2), bone disorders (3), and ischemic cardiovascular diseases (4). One feature common to all these conditions is the involvement of tissue regeneration and remodeling in their termination phase.

**Figure 1:**
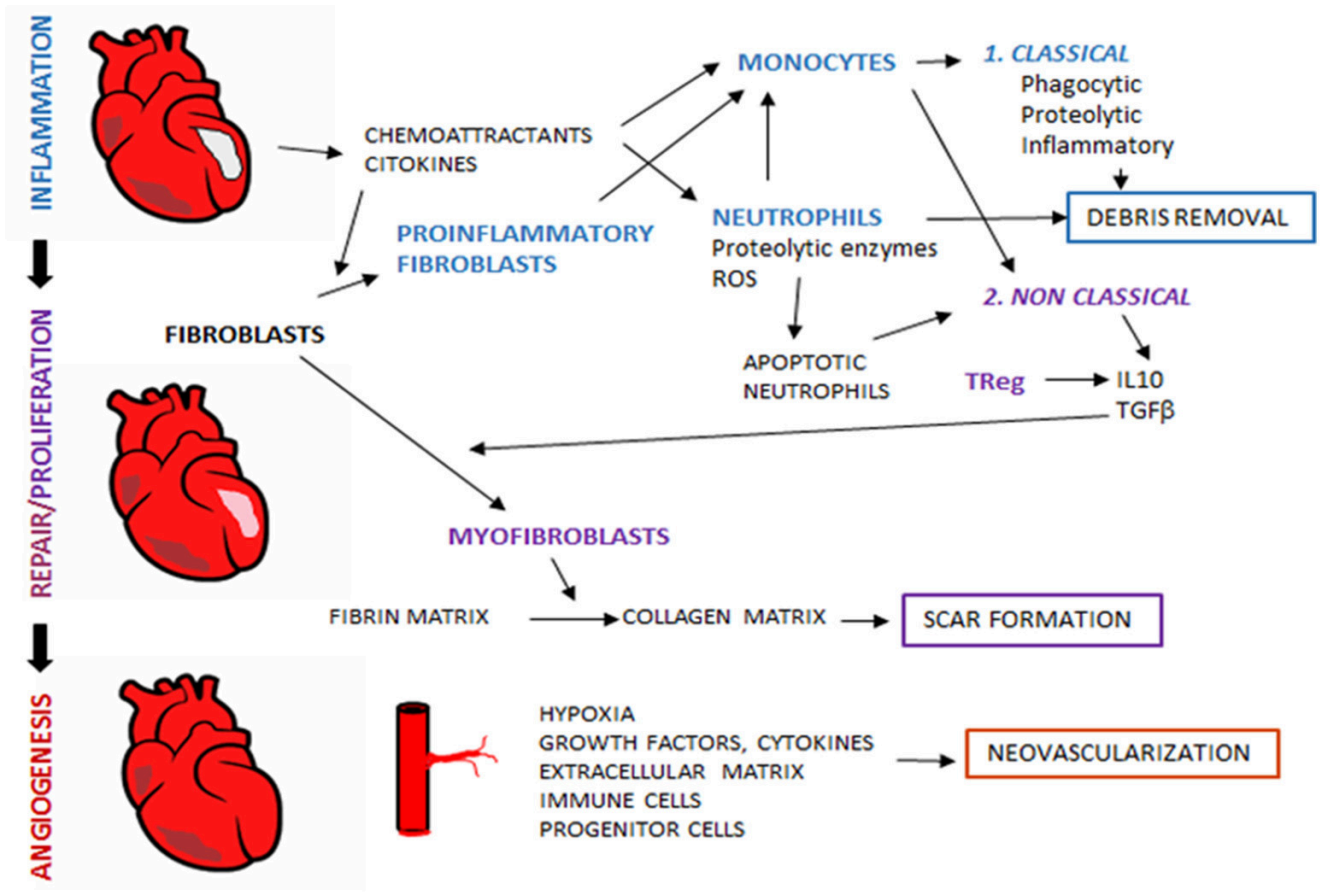
Tissue regeneration after myocardial infarction. After myocardial ischemia, remodeling process take place in order to reestablish tissue properties and myocardial function. During remodeling, sequential interdependent phases occur, which include inflammation, proliferation and repair. Al later stages, angiogenesis restores blood flow to ensure adequate tissue regeneration. Immune system is an essential player in tissue remodeling, and acts through the coordinated action with other non-immune cellular components and released mediators.

Myocardial infarction (MI) is the most common cause of early death in adults worldwide (5). It results from an imbalance between oxygen supply and demand, usually due to the occlusion of a coronary artery. Despite success in reducing early mortality, late morbidity and mortality in the form of heart failure (HF) are still an unmet clinical problem. HF after MI is the consequence of a non-optimal myocardial repair. After ischemia, a step-by-step myocardial remodeling takes place involving two main phases: an inflammatory phase, where recruited inflammatory cells clear the wound of tissue debris and prepare the tissue for the next phase, and the proliferative and reparative phase, where inflammation resolves, angiogenesis is induced, fibroblasts activated, and fibrosis takes place with scar formation (6). These highly orchestrated phases may seem to occur separately, but they actually overlap with a coordinated interplay between immune cells, fibroplasia and angiogenesis in order to provide an efficient microvascular perfusion of the remodeled heart, thus ensuring its subsequent repair and functionality (6) (Figure 1). Throughout the last decades, researchers have made efforts to promote myocardial repair by acting at different steps of the process, as is the case of angiogenesis (7). Indeed, the promotion of angiogenesis in MI patients seeks to improve the microcirculation by promoting new capillary and collateral arterial vessel formation, thus rescuing the myocardium at early stages post-MI and preventing long-term left ventricular remodeling and transition to HF (8–10).

Extracellular vesicles (EVs) are membranous vesicles involved in intercellular communication, released to the extracellular environment by different types of living cells under normal or pathological conditions. They can be classified into three main groups based on their biological origin, size and composition: microvesicles, apoptotic bodies and exosomes. Exosomes, here referred to as small EVs, are nanosized (50–150 nm) EVs of endocytic origin (11), which may act as a vehicle to transfer bioactive cargoes (proteins, lipids or nucleic acids) from one cell to another, either allowing or enhancing functional communication between cells (12, 13). Loading of these molecules into small EVs is not a random process; on the contrary, these vesicles are enriched with specific contents, thus revealing the existence of sorting mechanisms that selectively regulate small EVs load (14). Uptake of small EVs is mediated by direct interaction. Binding of small EVs to the plasma membrane of recipient cells is mainly driven by adhesion molecules involved in cell-to-cell interaction, but there are other small EVs molecules that specifically act as receptors for their selective binding and engulfment. To deliver their cargo, small EVs may either fuse with plasma membrane of recipient cells or be internalized by endocytosis, pinocytosis or phagocytosis (15).

Small EVs participate in a number of biological processes, which include organogenesis (16), vascular regulation (17) and immune responses (18). In these processes, cells from different origin release small EVs to generate an intricate signaling network, which reciprocally regulates cellular functions. In this review, we offer a perspective on the interaction between immune system and angiogenesis, and examine the functional role and potential application of immune system-derived small EVs in the promotion of neovascularization after MI.

## Interplay between immune response and angiogenesis in tissue remodeling after MI

### Immune response in the infarcted myocardium

During inflammatory phase, the first cells recruited to the injured site are neutrophils, which migrate in response to signals as damage-associated molecular patterns (DAMPs), cytokines, chemokines, endogenous lipid mediators (prostaglandin E2, leukotriene B4), histamine and complement components (19–21). Emigrant neutrophils release proteolytic enzymes and reactive oxygen species at their arrival to the infarcted site and damage local cells (22). Another key role of neutrophils in cardiac repair is the release of chemotactic factors such as azurocidin, IL-37 and cathepsin G that recruit and accumulate splenic monocytes in the infarcted myocardium (21, 23, 24). Two monocyte populations have been described in ischemic myocardium of both mice and human: an inflammatory subset Gr1+CCR2+ CX3CX1^lo^(Gr1^high^), and the resident monocyte subset with a less inflammatory function Gr1− CCR2− CX3CR1^hi^Gr1^low^, corresponding to classical human monocytes CD14^hi^CD16− and nonclassical monocytes CD14^lo^CD16+, respectively (25). Classical monocyte subset is recruited in the early phase of MI through the activation of the MCP-1/CCR2axis. These cells display a phagocytic, proteolytic and inflammatory function, by digesting the damaged tissue and clearing cellular debris at the injury site (26) (Figure 1).

After this phase of degradation and digestion of injured cells, new subsets of immune cells intervene, such as non-classical monocytes (Gr1^low^) with anti-inflammatory properties, T-cell subpopulations as regulatory T cells (TRegs), and macrophages with anti-inflammatory phenotype which secrete transforming growth factor (TGF)-β and IL-10. These cellular effectors and molecular signals repress inflammation and enhance heart healing through myofibroblasts accumulation, angiogenesis and collagen deposition (27) (Figure 1).

As soon as necrotic cells and debris are removed by the inflammatory cells, cells that play a crucial role in the first phase of regeneration are then replaced by resident or newly recruited Gr1low monocytes, lymphocytes and mast cells that will coordinate the remodeling of the myocardium. In fact, neutrophils have no longer an important role in the proliferation phase and despite DAMPs, proinflammatory cytokines, hypoxia and acidosis survival signals, neutrophils are short-lived cells that rapidly undergo cell death (19, 28). Indeed, late-stage and apoptotic neutrophils are essential for the end of the inflammatory phase, as their transmigration is slowed down by the release of pro-resolving mediators (such as lipid mediators, annexin A1 and lactoferrin) and their phagocytosis by macrophages is enhanced both by these mediators and by the expression of eat-me signals (19, 21). Phagocytosis of apoptotic neutrophils induces a pro-resolving M2 phenotype in macrophages and the subsequent secretion of anti-inflammatory and profibrotic cytokines such as IL-10 and TGF-β, which suppress inflammation and enhance tissue repair (29) (Figure 1).

Despite the well-established time frame of events during cardiac remodeling after MI, a subset of cases shows a late progressive ventricular dilatation and HF mainly due to a chronic inflammation state of the myocardium (30). Accordingly, it has been recently described that in chronic ischemic HF, there is local and systemic expansion of proinflammatory macrophages, dendritic cells (DC) and T cells with an impaired left ventricular remodeling (31).

### Angiogenic response in the infarcted myocardium

Angiogenesis is a highly orchestrated process that starts early after inflammatory phase of MI. The main players in hypoxia-driven angiogenesis are hypoxia-inducible factors (HIF). They are expressed in cardiomyocytes, endothelial and inflammatory cells early after MI, and may remain stable up to several weeks after MI (32). Both HIF-1α and endothelial-specific HIF-2α target proangiogenic genes such as VEGF (33), cyclooxygenase-2 (COX2) (34), the progenitor cell mobilizing chemokine Stromal Cell-Derived Factor 1-alpha (SDF1-alpha or CXCL12) (35), angiopoietins and others. Notably, several of these HIF-inducible genes also play an important role in inflammation, indicating a parallelism between inflammation and angiogenesis in ischemic-tissue remodeling.

A number of growth factors are implicated in angiogenesis promotion in the ischemic heart. Hence, an important increase of VEGF and its receptor VEGFR2 takes place in the viable border zone of myocardiocytes and in the area of forming vessels, respectively (36, 37). VEGF-B may also play a role in the induction of prosurvival signals that ensure the presence and maturation of new vessels in the ischemic myocardium, by providing anti-apoptotic signals in endothelial and mural cells (38). Similarly, PDGF-A and -D expression increases in the periphery of infarcted area, in parallel with the development of angiogenic process (39).

Different types of progenitor cells may act as a substrate for generation of new cells in angiogenesis in MI. Several studies have demonstrated proangiogenic and therapeutic effect of endothelial progenitor cells (EPCs) in experimental models of MI; however, the mechanisms involved are not well understood yet (40). Thus, although EPCs differentiate into mature ECs, there is growing evidence that their paracrine activity could play an important role in the injured endothelium repair (41). In a mouse model of MI, ischemic hearts injected with c-kit+ cardiac stem cells (CSCs) display higher angiopoietin-like 2 expression and staining for EC marker PECAM-1 in peri-infarct areas, suggesting that c-kit+ cells can differentiate into ECs and promote angiogenesis *in vivo* in these hypoxic sites (42). Other studies support this function of CSCs on vascularization recovery after MI, not only through differentiation into ECs, but also in a paracrine manner by promoting the secretion of multiple pro-angiogenic growth factors as VEGF, PDGF and HGF. (43). Mesenchymal stem cells (MSC) and adipose tissue-derived stem cells have also shown a proangiogenic potential after MI in experimental studies (44). MSC enhance several key processes in angiogenesis by releasing paracrine factors that stimulate vessel formation, differentiating into endothelial of vascular smooth muscle cell lineage, and acting as perivascular cells (45). The proangiogenic function of MSC has been related to both the release of soluble factors and miRNA, notably miR-146a, which increases VEGF secretion in MSCs leading to a reduction in fibrosis and enhancement of left ventricular ejection fraction (45, 46).

### Immune regulation of angiogenic response

**Figure 2:**
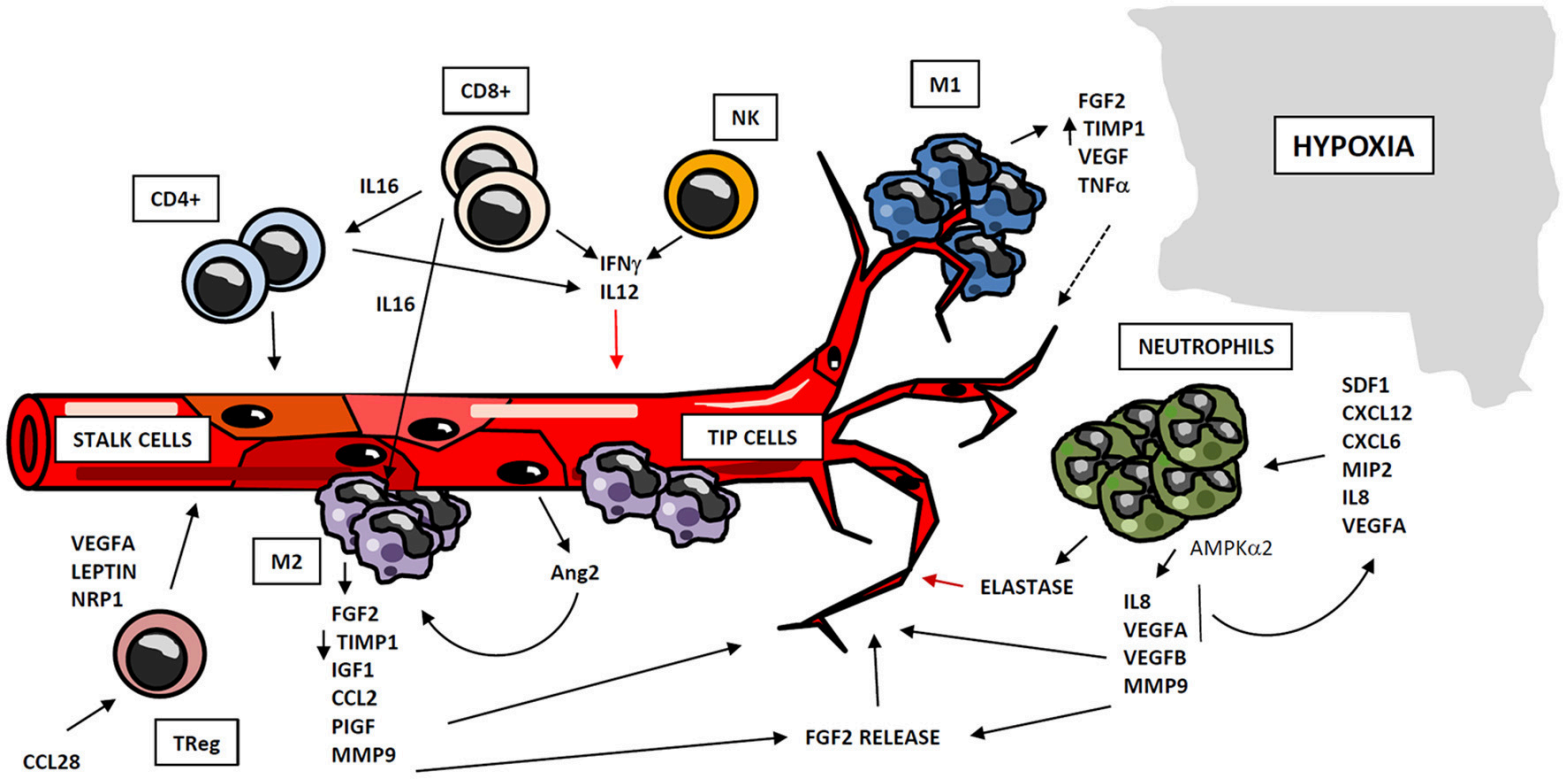
Regulation of angiogenesis by cellular compartment of immune system. Immune system participates in angiogenesis development after myocardial ischemia, through cellular and soluble components. Myeloid and lymphoid cells may operate as positive or negative regulators of angiogenesis, through the secretion of cytokines and soluble factors which act at different stages of the process. Monocytes act in two subsequent waves of infiltration, composed respectively by M1and M2-like cells. M1 macrophages co-localize with endothelial tip cells and display a mild pro-angiogenic phenotype, whereas M2 cells are located close to EC anastomosis, and possess potent pro-angiogenic properties. Neutrophils are recruited to hypoxic areas by chemoattractant cytokines, and accumulate in so-called “angiogenic hotspots” at vascular tips. Neutrophils mainly exert proangiogenic actions by the production of soluble factors and metalloproteinases, although these cells may act as negative regulators of this process, trough elastase release. Lymphocytes also play an important role in angiogenesis, either directly by the secretion of pro- and anti-angiogenic mediators, or by regulating the activity of other cell types as macrophages and different lymphocyte subsets.

As mentioned above, inflammation and angiogenesis are closely inter-related processes. The immune system handles, along with other cells and mediators, the correct and efficient repair of the damaged heart, and more specifically an adequate angiogenesis development. This is achieved by the concerted action of cellular and molecular components (summarized in Table 1), which display pleiotropic functions that modulate their respective activities (67) (Figure 2).

**Table 1:** Function of interleukins and chemokines in angiogenesis regulation.

**Cytokine**	**Function**	**Mechanism**	**References**
Il-1β	Pro-angiogenic	Induction of VEGF production by tumor and stromal cells	(47)
IL-2	Pro-angiogenic	Increase of ROS levels and phosphorylation of Akt in ECs	(48)
IL-6	Pro-angiogenic	Upregulation of key angiogenesis-associated genes	(49)
IL-8	Pro-angiogenic	Promotion of Akt and GSK-3βser9 expression, inducing proliferation and inhibiting apoptosis in ECs	(50)
IL-16	Pro-angiogenic	Recruitment of pro-angiogenic T CD4+ and mononuclear cells	(51)
IL-17	Pro-angiogenic	Stimulation of VEGF production via STAT3 signaling pathway	(52)
IL-19	Pro-angiogenic	Promotion of EC proliferation, migration and tube-like formation Induction of M2-phenotype macrophage polarization, synthesis of VEGF-A in macrophages and reduction of IL-12 expression	(53)
IL-12	Anti-angiogenic	Arrestment of EC cycle Induction of activated NK cells infiltration	(50) (54)
IFNγ	Anti-angiogenic	Reduction of VEGF and downregulation of Dll4 in ECs Reduction of SDF-1/CXCR4 expression in ECs	(55) (56)
CCL2	Pro-angiogenic	Recruitment of macrophages with proangiogenic phenotype	(57)
CXCL1	Pro-angiogenic	Enhancement of ERK1/2 signaling in ECs, leading to a EGF expression and secretion	(58)
CXCL6	Pro-angiogenic	Induction of EC chemotaxis Attraction of neutrophils loaded with MMP-9	(59)
CXCL12	Pro-angiogenic	Enhancement of EC proliferation, migration, and adhesion via activation of the CXCR4 pathway	(60)
CX3CL1	Pro-angiogenic	Recruitment of CD11b+CX3CR1+ proangiogenic macrophages	(61)
CXCL4	Anti-angiogenic	Inhibition of EC adhesion to matrix proteins	(62)
CXCL9	Anti-angiogenic	Inhibition of blood vessel formation by interacting with VEGF and preventing its binding to ECs	(63)
CXCL10	Anti-angiogenic	Antiproliferative effect on EC as result of its affinity for GAGs and the resultant displacement of growth factors from the cell surface	(64)
CXCL14	Anti-angiogenic	Inhibition of angiogenic ligands (IL-8, bFGF) by direct interaction, avoiding their binding to high affinity receptors	(65)
CXCL11	Receptor dependent action	Signaling through CXCR3 has been found to have anti-angiogenic effects, while signaling through the CXCR7 is most likely to be pro-angiogenic.	(66)

Cells of myeloid lineage are the best described immune regulators of angiogenesis. Specifically, monocytes/macrophages exhibit well-know functions in vessel formation (68). A number of studies suggest that recruitment of monocytes to ischemic tissues takes place in two waves of infiltration: the first wave attracts monocytes with M1 inflammatory phenotype, which are subsequently replaced by a second wave, composed of pro-angiogenic and regenerative M2-like macrophages (69, 70). Macrophage-secreted Semaphorin 4A has been involved in angiogenesis occurring after ischemic injury in the heart (71). M2 macrophages promote neovascularization through the release of proangiogenic factors as FGF-2, insulin-like growth factor-1 (IGF-1), monocyte chemotactic protein-1 (CCL2), placental growth factor (PIGF) (72), and proteases as MMP-9 (73). M1 macrophages may have a positive role in angiogenesis as well, although to a different extent: both M1 and M2 macrophages produce MMP-9, but M2 macrophages display a reduced expression of tissue inhibitor of metalloproteinase 1 (TIMP-1), so their angiogenesis-inducing capacity is higher than that of the M1 macrophages (74). Additionally, M1 macrophages co-localize with endothelial tip cells and M2 macrophages with cell anastomosis sites, thus revealing a potential interaction of M1 phenotype with tip cells and a participation of M2 macrophages in cell fusion (75, 76). Therefore, macrophage-EC contact seems to be important in vascular morphogenesis. Furthermore, paracrine signaling may participate in macrophage pro-angiogenic functions. Activated ECs secrete angiopoietin-2 (Ang2), which binds neighbor Tie2-expressing monocytes/macrophages, enhancing their angiogenic potential (77). All these findings point to monocytes/macrophages as key regulators of angiogenesis, suggesting that there is a coordinated participation of both subsets of macrophages in vessel formation. Hence, M1 macrophages sustain the initial tissue inflammation, remove damaged tissue and finally begin angiogenic process and vessel growth. They are subsequently replaced by M2 macrophages, which display more pro-angiogenic capacities (Figure 2).

Neutrophils play an important role in angiogenesis through different mechanisms. One is regulation of vascular repair through AMP-activated protein kinase α2 (AMPKα2), a subunit of AMPK protein, which promotes survival of neutrophils in ischemic tissues as well as generation of pro-angiogenic factors (such as VEGFA and VEGFB) (78). Aside, ischemic-infiltrating neutrophils produce TIMP-1-free proMMP-9 that proteolytically induces functional angiogenic activity (74) through bioactive FGF-2 release (79). Furthermore, it has been demonstrated that VEGFA released under ischemic conditions promotes the recruitment of a specific subset of circulating CD49d+ VEGFR1^high^ CXCR4^high^ neutrophils with high MMP-9 expression levels, to facilitate rapid angiogenesis at hypoxic areas (80, 81). Other stimuli lead to neutrophil recruitment. Thus, CXCL1/macrophage inflammatory protein-2 (MIP-2) induces neutrophil recruitment and release of active VEGFA, which in turn activates the angiogenic cascade in tissues (82). Besides, IL-8/CXCL8 induces recruitment of neutrophils, which secrete VEGF and more IL-8, acting as a paracrine feedforward signal that amplifies the angiogenic effect of this cell type. Infiltrating neutrophils accumulate in “angiogenic hotspots,” specifically localized at the tips of the sprouting vessels; and this recruitment is directed by SDF-1 (CXCL12), a chemokine produced in the proangiogenic niche (83). However, some evidences suggest that neutrophils could secrete factors that restrain the angiogenic process. For instance, neutrophils release neutrophil elastase, a serine proteinase that cleaves plasminogen into angiostatin (fragments one to three), which in turn inhibits VEGF and FGF-2-mediated EC proliferation (84) (Figure 2). Despite the above mentioned proangiogenic characteristics of neutrophils, they are one of the major mediators of microvascular dysfunction after MI, and promote ischemia reperfusion injury and no-reflow phenomenon (10, 85).

Lymphocytes have been shown to mediate neovascularization as well. B lymphocytes infiltrating tumors contribute to angiogenesis through STAT3-mediated production of pro-angiogenic factors such as MMP-9 or CCL2 (86). Likewise, both CD4+ and CD8+ T lymphocytes play an important role in this process, together with other subpopulations as TReg and Tγδ cells. CD4+ T lymphocyte-deficient mice demonstrate impaired post-ischemic neovascularization, which is rescued by CD4 reconstitution (87). Similarly, CD8+ T lymphocyte-deficient mice also display defective blood flow recovery after hindlimb ischemia, which recovers upon CD8+ infusion through IL-16-mediated CD4+ and mononuclear cell recruitment (51). However, lymphocytes may participate also in restraining angiogenesis. It has been shown that lymphocytes (mostly CD4+)-EC crosstalk mediates IL-12-dependent inhibition of angiogenesis by reducing expression and activity of MMP-9 *in vitro* (88, 89); and in a mouse model of lung ischemia, neovascularization is limited by both CD8+ and CD4+ effector T cells through interferon gamma (IFNγ) secretion (90) (Figure 2). Likewise, Tγδ cells are able to promote angiogenesis in tumors by differentiating into IL-17 producing cells (91). Additionally, in a model of oxygen-induced retinopathy, acute retinal ischemia induces TReg recruitment, although this accumulation is not enough to completely prevent vascular damage by itself; but expanding TReg population by administrating IL-2/anti-IL2 monoclonal antibody complexes to mice, TRegs are able to reduce retinopathy (92). Furthermore, Natural Killer (NK) cells are involved in angiogenesis regulation as well. In a model of murine hindlimb ischemia, depletion of NK1.1-expressing cells led to defective perfusion recovery and collateral blood vessels growth (93). Moreover, IFNγ synthesis is increased in tumor-infiltrating NK and CD4+ T cells, and this may be responsible for production of tumor necrosis factor superfamily-15 (TNFSF15) by ECs, a cytokine that inhibits their proliferation and differentiation (94). In conclusion, lymphocytes may act as positive or negative regulators of neovascularization, frequently through the secretion of cytokines and soluble factors that operate in different steps of the angiogenic process.

## Small EVs in angiogenesis

**Figure 3:**
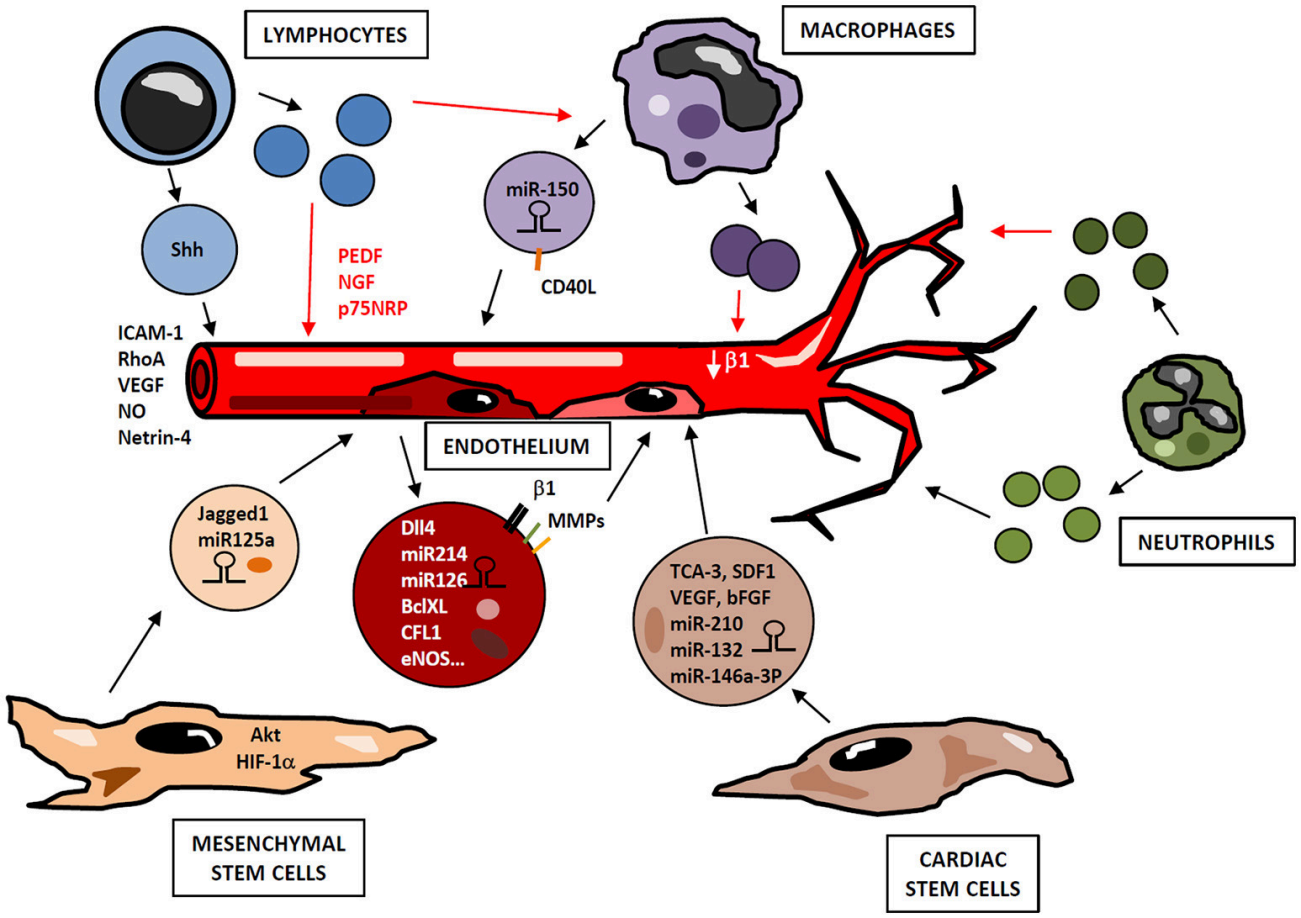
Small EVs in angiogenesis regulation. Cells from immune (lymphocytes, macrophages, neutrophils) and non-immune (cardiac and mesenchymal stem cells, endothelial cells) compartments release small EVs containing bioactive cargos (miRNAs, mRNAs, cytokines, signaling molecules), which modulate endothelial functions in a paracrine manner, positively or negatively regulating neovascularization. Depending on their activation state, lymphocytes may produce small EVs with either pro- or antiangiogenic effect. Similarly, small EVs derived from activated macrophages and neutrophils display different functional properties. Thus, a highly complex network of vesicles with potential opposite functions may be released during angiogenic process in response to immune cell activation and microenvironmental conditions.

Cells belonging to either non-immune or immune compartments contribute to angiogenesis development through the delivery of small EVs, which act in a paracrine manner and trigger functional responses in neighboring cells (Figure 3).

ECs-derived microvesicles contain β1-integrin, MMP-2 and MMP-9; and their addition to human umbilical vein endothelial cells (HUVEC) stimulates formation of capillary-like structures *in vitro* (95). Interestingly, Dll4 is incorporated into EC-derived small EVs and can be transferred from one cell to another in a paracrine manner, thus inhibiting Notch receptor signaling and enhancing vessel growth and branching (96). Likewise, EC-derived small EVs contain miRNAs such as miR-214, which promotes angiogenesis both *in vitro* and *in vivo* by suppressing cell cycle arrest (97). miR-126-3p has also been identified as an important player in endothelial repair cell migration, proliferation and regeneration by inhibiting EVH1 domain-containing protein1 (SPRED1) and promoting Ras/MAPK signaling (98). Furthermore, small EVs derived from human EPCs induce angiogenesis by the horizontal mRNA transfer of genes such as BCL-XL, CFL1, CTNNB1, EDF1, MAPKAPK2, and eNOS (99) (Figure 3).

MSCs are another source of small EVs with pro-angiogenic potential (Figure 3). MSC-derived small EVs induce the formation of new vessels in the infarcted myocardium, inhibit cardiac remodeling and preserve the ejection function of the damaged heart (100, 101). In addition to this, a comparative study of small EVs and MSCs therapy revealed that small EVs injection was more effective and improved cardiac fibrosis, inflammation and cardiac performance (102). Regarding angiogenesis, *in vivo* studies have shown that small EVs from MSCs overexpressing Akt enhance neovascularization with a subsequent improvement of LVEF (103). Similar results were obtained with MSCs overexpressing HIF-1α. Indeed, these small EVs contain Jagged1, and induce angiogenesis in ECs both *in vitro* and *in vivo* by activating Notch signaling (104). In a renal ischemia-reperfusion model, treatment with umbilical cord MSC-derived small EVs improved capillary density by inducing VEGF elevation in a HIF1α independent manner (105). Small EVs derived from MSCs also transfer miRNAs as miR-125a to ECs, thus promoting angiogenesis (106, 107).

Other adult stem cells, as CSC, secrete small EVs enriched with cytokines and chemokines (TCA-3, SDF1), vascular growth factors (VEGF, erythropoietin, bFGF, osteopontin, SCF) and cardiac differentiation factors (Activin A, Dkk homolog-1, TGF-β) (108). In addition, CSCs derived small EVs analysis revealed high levels of miR-210, miR132 and miR-146a-3p that enhance anti-apoptotic and pro-angiogenic activity (Figure 3). This seems to be mediated by activation of their downstream targets such as ephrin A3, PTP1b and RasGaP-p120, that eventually leads to augmented cardiac function (109). Similarly, a study showed that bone marrow-derived CD34+ cells secreted small EVs with pro-angiogenic activity in ischemic cardiac tissue, mainly due to the delivery of pro-angiogenic microRNAs such as 130a and 126 (110).

Immune system-derived EVs originate from lymphocytes, monocytes/macrophages or neutrophils, and their selective cargo and surface biomolecules modulate neovascularization by different mechanisms. Several studies have been conducted to assess the effect of immune system-derived EVs in angiogenesis, however the specific role of small EVs in this process has not been exhaustively addressed. Lymphocytes constitute an important source of EVs that may exert pro- or anti-angiogenic effects depending on stimuli involved in their production (Figure 3). When lymphocytes undergo activation before apoptosis, they release proangiogenic microvesicles. Thus, *in vitro* experiments show that treatment of T cells with phytohemagglutinin, phorbol ester and actinomycin D induces production of microvesicles, so-called lymphocyte microparticles (LMPs), which lead to the formation of capillary-like structures. This pro-angiogenic effect is mediated by increasing cell adhesion and promoting expression of ICAM-1, Rho A and VEGF, through activation of Sonic Hedgehog (Shh) pathway (111). In a mouse model of ischemia-reperfusion, microvesicles from T lymphocytes carrying Shh are involved in restoration of endothelial function by NO production (112). Similarly, in a model of hindlimb ischemia, EVs harboring Shh promote post ischemic neovascularization by enhancing vascular density (113). HUVECs can internalize EVs from T lymphocytes, a process that modifies HUVEC gene expression profile, including modulation of some angiogenic-related genes as netrin-4, and increases tube length (114). Conversely, lymphocytes treated with actinomycin D without previous activation produce anti-angiogenic LMPs, through a mechanism not well-defined yet. Thus, these microvesicles suppress neovessel formation in rat choroidal explants by increasing mRNA levels of pigment epithelium-derived factor (PEDF), nerve growth factor (NGF) and p75 neurotropin receptor (p75NRP) (115). Likewise, in a model of corneal neovascularization, LMPs reduce angiogenesis and inhibit human ECs proliferation, survival and migration by inducing reactive ROS and interfering with VEGFR-2 pathway (116). Moreover, LMPs produced after treatment with actinomycin D alter the expression of pro-angiogenic factors in macrophages through CD36-mediated signal transduction pathways (117).

Microvesicles derived from monocytes and macrophages participate in angiogenesis regulation as well (Figure 3). Transfer of miR-150 to ECs by monocyte-derived microvesicles enhances neovascularization in *in vivo* and *in vitro* models by downregulating c-Myb (118) and enhancing ECs migration (119). In addition, CD40 Ligand+ (CD40L) microvesicles originated from atherosclerotic lesions stimulate EC proliferation and neovessel formation *in vivo*. Most of these microparticles express CD14 on their surface, suggesting that they are mainly of monocytic origin (120). However, the role of myeloid-derived small EVs in neovascularization is controversial. In an *in vitro* assay with HUVECs, small EVs derived from monocytes adhere to these ECs stimulating them and enhancing tube formation, but also inducing apoptosis (121). In another study, small EVs derived from macrophages modulate integrin-β1 trafficking by enhancing its internalization and degradation in ECs, thus impairing their migration (122). Furthermore, similar to LMPs after actinomycin D treatment, monocyte-derived EVs inhibit tube formation and migration in microvascular ECs through CD36 signaling. However, this inhibitory effect decreases when small EVs are isolated by differential centrifugation from other EVs, since small EVs-containing fraction inhibits EC migration to a lesser extent than small EVs-depleted EVs (123). In addition to monocyte/macrophage-derived EVs, EVs from DCs can also mediate neovascularization. DC-derived small EVs harbor active MMP-9 (124), a pro-angiogenic factor that acts in ECM remodeling to allow neovessel formation.

Finally, neutrophils also secrete small EVs in a stimulus-dependent manner. When stimulated with N-Formylmethionyl-leucyl-phenylalanine (fMLP), they secrete small EVs with anti-inflammatory potential, which up-regulate EC-protective factors as ANGPTL4 or CD55. On the contrary, neutrophils pre-incubated with a HUVEC monolayer before addition of fMLP produce a population of small EVs that display a pro-inflammatory, anti-apoptotic phenotype (125).

This experimental evidence highlights that leukocyte-derived EVs can generate both pro- and anti-angiogenic effects, which may be due to different cellular origin or microenvironment. Moreover, diverse EVs subpopulations, such as small EVs and other microvesicles, seem to play distinct roles in promoting or inhibiting angiogenesis. Such differences may rely either on diverse cargoes or on specific expression patterns of membrane molecules in these EVs subsets. A detailed study of mechanisms that regulate these functional differences may help to design therapies that profit from small EVs-mediated paracrine effect to foster tissue repair and angiogenesis in ischemic cardiac tissue.

## Small EVs treatment in the infarcted myocardium: novel therapy approaches

Current therapies of MI mainly focus on macrovascular reperfusion of the infarcted myocardium with thrombolytic therapy, primary percutaneous coronary intervention and coronary artery bypass grafting. Early reperfusion of the culprit artery has proven to be the most successful therapy in reducing mortality, being the gold standard (126). Indeed, early mortality after MI has decreased over the last decades, from 20% in the late 80's to approximately 5–7% in contemporary series (127, 128). However, delayed HF secondary to inadequate cardiac remodeling is still a challenge in therapeutic management of MI. Angiogenesis plays an essential role in cardiac repair and regeneration after ischemic heart disease; therefore, therapeutic angiogenesis has been considered as a promising therapy for patients suffering MI. Over the past decades, a variety of strategies to promote therapeutic angiogenesis has been evaluated. However, there is still a need to come up with a balanced and targeted therapy that ensures an optimal restoration of blood supply into the damaged myocardium in order to enhance a correct tissue healing and prevent HF.

Both preclinical models (129, 130) and early human clinical trials (131) demonstrated promising outcomes with strategies consisting of administration of proangiogenic factors as VEGF; however larger clinical trials using individual proangiogenic factors showed mixed results (132, 133), thus indicating that delivery of a single growth factor was not sufficient to support a complete and mature angiogenesis in these patients.

After major failure of proangiogenic growth factors to show any benefit in clinical trials, cell transplantation quickly became the next frontier therapy for promoting angiogenesis and cardiac regeneration (134, 135). Several stem cells have been used as cellular sources to produce new cardiac cells such as endothelial mesenchymal stem cells (EMCs), MSCs, and cardiac progenitor cells (CPCs), showing safety and promising results (136, 137). Several clinical trials have been launched to probe therapeutic utility of progenitor cells from different sources in MI treatment, with different outcomes (138–143). However, these results could not be fully assigned to the exogenous cells, as it seems that in mouse models nearly 90% of newly formed cardiomyocytes and ECs are of endogenous origin and do not come from the exogenous cells (144). Thus, paracrine factors secreted by cell transplantation appear to mediate endogenous repair, through modification of functions of neighboring cells. In this line of evidence, a recent study demonstrated that intracoronary injection of cardiac-derived stem cells (CDC)-secreted small EVs reduced the number and modified the polarization state of CD68+ macrophages in the infarcted myocardium, leading to increased expression of anti-inflammatory genes such as *Arg1, IL4ra, Tgfb1*, and *Vegfa*. In this study, RNA-Seq analysis of macrophages primed with CDC small EVs showed enhanced levels of miR-181b, which targets protein kinase C δ (145). Another study showed that the most abundant RNA species in CDC small EVs was a Y RNA fragment (EV-YF1), which was transferred to macrophages. This transfer induced transcription and secretion of the anti-inflammatory IL-10, thus promoting protection of cardiomyocytes from oxidative stress and reducing infarct size (146).

These findings led to the idea that small EVs secreted by different cell populations could become a potential therapeutic tool to improve cardiac function after MI through tissue remodeling and more precisely by promoting angiogenesis in the damaged heart.

Small EVs have a biological signature that mimics the phenotype of the cells that produced them (15). Hence, there is growing interest in small EVs and their application in the clinical context. Indeed, they play an important role as transporters of paracrine factors in angiogenesis, immune regulation, and tissue regeneration (147). This has led to the notion that small EVs could become a delivering system for regenerative therapeutic factors. Thus, administration of CDC small EVs has shown the same benefits in infarcted hearts of animal models with enhancement of angiogenesis and promotion of cardiomyocytes survival and proliferation (148). Moreover, despite concerns regarding standarization of isolation procedures, small EVs offer clear advantages over cell-based therapies (summarized in Table 2). Hence, these vesicles have small size and low immunogenicity, are nontoxic, permeable to physiological barriers and stable in blood (149–151). Indeed, they can be preloaded with specific therapeutic agents as well as protected from degradation and deactivation. Protein changes on their surface ensure affinity to specific cell types, thus enhancing efficient and selected target cell communication (152). Cell modification is generally achieved either by hijacking biosynthesis to favor the production of specific endogenous material or by delivering exogenous species to the cytoplasmic membrane (158). Main current small EVs engineering strategies are summarized in Table 3.

**Table 2:** Main advantages of Small EVs over cell-based therapies.

	**Cells**	**Small EVs**	**References**
Size	+ +	–	(149–151)
Immunogenicity	+ –	–	
Permeability to physiological barriers	+ –	+	
Loading with specific therapeutic agents	–	+	(152)
Targeting to selected tissues/cell types	+ –	+	
Safety	+ –	+	(153)
Storage & shipping	Complex (cryogenic)	Simple (regular freeezing/lyophilization)	(154, 155)
Complexity of clinical application	High (thawing, shear stress upon injection)	Low	(156, 157)

**Table 3:** Overview of main strategies for small EVs engineering.

**Type**	**Subtype**	**Mechanism**	**Advantages**	**Limitations**	**References**
Cell modifications	Genetic modification	Transgene expression induction in the parent cell for further small EVs loading that include specific surface receptors	– Imaging visualization of small EVs distribution after small EVs systemic administration. – Ability to target specific organs	Requires high knowledge on vesicle loaded protein molecular biology.	(159) (160) (161)
	Metabolic labeling	Integration in the proteome, lipidome, genome and glycome of exogenous metabolites	– Few side effects – Applications for drug conjugation, cell killing and surface gelation.	– Biomolecules modification in the entire cell – Poor expertise on bio-orthogonal chemistry	(162) (163) (164)
	Uptake of Exogenous delivery	Introduction of exogenous material to the cell by incubation		– Dependent on the amount of material delivered to the cell – Long-time exposures – High concentration of nanoparticles	(165) (166)
		Theranosomes production	*in vivo* magnetic targeting.	Dependent on macrophage phagocytosis	(167) (168)
Post-isolation small EVs modifications	Active loading of small EVs	Membrane molecules attachment by covalent bonds using “click chemistry”	– Rapid and efficient – No modification on size, structure and function of small EVs.	Small EVs functionality impairment by surface chemical modifications	(169)
		Extrusion	High loading efficiency	Membrane alteration	(170) (171)
		Conjugation of native and non-native small EVs surface receptors with magnetic nanoparticles	– High effectiveness and specificity – Small EVs functionality modification – Elimination of undesired vesicles implicated in pathological situations	Synthetic challenge	(172) (173)
		Small EVs membrane loading with hydrophilic cargo after saponin incubation	Incorporation of multiple small lipophilic drugs	– Technical difficulties – Toxicity	(174) (170)
		Electroporation or chemical transfection of siRNA, small molecule drugs and superparamagnetic iron oxide nanoparticles.	High efficiency	Disruptive strategy that endangers the integrity and functionality of the EV	(175) (161) (176)
	Passive loading of small EVs	Incubation with drugs	Simple technique No membrane compromise	Low efficiency	(171) (177)
		Incubation of donor cells and further secretion of drug loaded small EVs		Possible drug citotoxicity	(177)

Different strategies have been developed to enhance cardiac capture and retention of small EVs: Systemic administration of small EVs conjugated with a synthetic cardiac homing peptide using a DOPE-NHS linker, shows reduced fibrosis and scar size as well as increased cellular proliferation and angiogenesis (178). Another attempt to target small EVs to the heart has been performed using a fusion protein comprising synthetic cardiac-targeting peptide (CTP)-Lamp2b, which is expressed in small EVs membrane. This construct is expressed in small EVs-producing cells through transfection, and stabilized in small EVs membrane by the attachment of glycosylation sequences. Delivery of these modified small EVs to the heart has shown significant but mild results (179).

Small EVs derived from immune system have been also engineered both endogenously and exogenously through modifications aimed at improving their therapeutic potential, usually modifying their cargo. Small EVs derived from EL-4 cells (mouse lymphoma cell line) were passively loaded with anti-inflammatory curcumin, which led to increased drug stability and delivery in a lipopolysaccharide (LPS)-induced septic shock mouse model (174) and a LPS-induced brain inflammation model (180). Additionally, siRNA against α-synuclein gene, which generates pathological aggregates in Parkinson's disease, was loaded by electroporation into murine DCs small EVs; these small EVs specifically target brain by expressing rabies virus glycoprotein peptide (RVG)-Lamp2, where a downregulation of α-synuclein was achieved (181, 182). Likewise, small EVs derived from M12.4 murine B cell line were loaded with miR-155 by electroporation and delivered to macrophages, thus suppressing LPS-induced TNF-α production (183).

Both endogenous small EVs function in tissue repair and angiogenesis, and the feasibility of small EVs modification to optimize specific cargo loading and tissue targeting, highlight the potential of these EVs in proangiogenic MI therapy. However, further definition of the appropriate cellular source and characterization of specific functional characteristics of these vesicles is needed before their clinical application. Thus, pleiotropic effects on angiogenesis of small EVs derived from different types of immune cells with diverse activation states, may represent a challenge for optimizing the production of a suitable subset of vesicles. Similarly, most preclinical studies have been conducted using cell lines; and a clear advantage in terms of therapeutic efficacy, biosafety, and clinical application of small EVs derived from cell lines over those isolated from more physiological sources, has not been established yet.

## Concluding remarks

When myocardial ischemia occurs, a tissue remodeling process takes place that involves sequential interdependent stages. These include an inflammatory phase and subsequent proliferative and reparative phases, where inflammation terminates and angiogenesis is induced. Immune system components are major regulators of cardiac tissue remodeling, which is especially remarkable in the case of angiogenesis. Some of these regulatory functions may be carried out through the release of small EVs loaded with bioactive molecules that act in a paracrine manner in adjacent cells. Likewise, benefit associated to cell therapy is in part mediated by paracrine factors including small EVs. Indeed, small EVs features demonstrate a significant advantage over cell-based therapies due to their stable physiology, their low immunogenicity and safety profile, their storage and application characteristics, and their ability to deliver therapeutic cargoes selectively to the damaged cardiac cells. These characteristics unveil the unique properties of small EVs as tools for the development of selective cell-free therapies to promote angiogenesis during tissue regeneration in the infarcted myocardium. Further investigation is needed to identify the optimum immune cell source and isolation conditions required to obtain small EVs adequate for successful clinical application in myocardial remodeling.

## Author contributions

SS-A, AA-S, FS-M, and AA had scientific discussion for this work and wrote the manuscript.

### Conflict of interest statement

The authors declare that the research was conducted in the absence of any commercial or financial relationships that could be construed as a potential conflict of interest.
